# Integrated PbTe Quantum Dots for Two-Color Detection in II–VI Wide-Bandgap Diodes

**DOI:** 10.3390/nano16010007

**Published:** 2025-12-19

**Authors:** Jakub M. Głuch, Michał Szot, Grzegorz Karczewski

**Affiliations:** 1 Institute of Physics, Polish Academy of Sciences, Al. Lotników 32/46, 02-668 Warsaw, Poland; jgluch@ifpan.edu.pl (J.M.G.); szot@ifpan.edu.pl (M.S.); 2 International Research Center MagTop, Al. Lotników 32/46, 02-668 Warsaw, Poland

**Keywords:** two-color detectors, infrared detectors, quantum dots, photodiodes

## Abstract

Quantum dots (QDs) composed of the narrow-bandgap semiconductor PbTe were incorporated into the depletion region of p–n junctions based on wide-bandgap II–VI semiconductors (p-ZnTe/n-CdTe). The heterostructures were grown by molecular beam epitaxy (MBE) on semi-insulating GaAs (100) substrates. The depletion region was engineered by depositing 20 alternating thin layers of CdTe and PbTe, then thermal annealing under ultrahigh vacuum. As revealed by cross-sectional scanning electron microscopy (SEM), the initially continuous PbTe layers transformed into arrays of zero-dimensional nanostructures, namely PbTe QDs. The formation of PbTe QDs in a CdTe matrix arises from the structural mismatch between the zinc blende and rock-salt crystal structures of the two materials. Electron beam-induced current (EBIC) scans confirmed that the QDs are localized within the depleted charge region between the p-ZnTe and n-CdTe layers. The resulting wide-gap diodes containing narrow-band QDs show pronounced sensitivity to infrared radiation in the spectral range of 1–4.5 μm, with a peak responsivity of approximately 8 V/W at a wavelength of ~2.0 μm and a temperature of 200 K. A red-shift in the cutoff wavelength when temperature decreases indicates that the infrared (IR) response is governed by band-to-band optical transitions in the PbTe QDs. In addition, the devices show sensitivity to visible radiation, with a maximum responsivity of 20 V/W at 0.69 μm. These results demonstrate that wide-bandgap p–n junctions incorporating narrow-bandgap QDs can function as dual-wavelength (visible and infrared) photodetectors, with potential applications in two-color detection and infrared solar cells.

## 1. Introduction

Lead telluride (PbTe) is a narrow-band-gap semiconductor with bandgap energies of about 0.32 eV at room temperature and 0.22 eV at 60 K [1]. It is well known for its strong mid-infrared photosensitivity. The main factors determining both the type of conductivity and the carrier concentration in PbTe are deviations from stoichiometry, which can be precisely controlled during growth or through post-growth treatments. This control enables the fabrication of p–n junction-based devices [2,3].

Cadmium telluride (CdTe), a II–VI semiconductor with a wide bandgap, is highly valued for its excellent optical properties in the visible spectrum, making it suitable for light detectors and efficient solar cells [4,5,6,7]. These properties include a very high absorption coefficient, on the order of 10^6^ cm^−1^, and a direct bandgap of 1.49 eV at room temperature, which closely matches the maximum of the solar spectrum. CdTe can be doped to exhibit either n-type or p-type conductivity, enabling the production of p–n diodes. To increase hole concentration, p-type CdTe layers are often combined with acceptor-doped Zn-containing materials, such as CdZnTe or ZnTe.

Structures made of both CdTe and PbTe can be effectively grown using epitaxial techniques, such as molecular beam epitaxy (MBE), because both materials are cubic semiconductors with very similar lattice constants (0.646 nm for PbTe and 0.648 nm for CdTe [8]), leading to a tiny lattice mismatch of about 0.3%. However, PbTe crystallizes in a rock-salt structure, while CdTe adopts a zinc-blende structure. This difference has significant consequences: (1) PbTe and CdTe are nearly immiscible, forming separate regions of pure PbTe and CdTe with well-defined interfaces; (2) the interfaces between PbTe and CdTe have a high density of dangling bonds that can trap free carriers from neighboring PbTe regions. This immiscibility has been used to create PbTe quantum dots with well-defined, sharp boundaries embedded in a CdTe matrix, which show strong infrared photo- and electroluminescence [8,9,10,11]. Conversely, CdTe quantum dots in a PbTe matrix have been studied through transport measurements to examine how antidots affect the thermoelectric performance of PbTe films [12,13]. Photoresistors based on PbTe/CdTe multilayers exhibit strong infrared photosensitivity up to room temperature, making them promising for high-temperature infrared detection applications [14,15,16,17].

This study aims to extend the previous investigation of p-ZnTe/n-CdTe diodes with PbTe nano-inclusions incorporated into their charge-depletion region [17]. The novelty of this work lies in the use of PbTe nano-inclusions in the form of zero-dimensional quantum dots (QDs), in contrast to our previous study, which focused on the influence of two-dimensional PbTe layers or multilayers on the performance and characteristics of wide-bandgap p-ZnTe/n-CdTe diodes. Our results demonstrate that incorporating PbTe QDs significantly broadens the optical sensitivity of wide-bandgap diodes, extending it into the mid-infrared region. Importantly, this enhanced infrared sensitivity persists at room temperature, highlighting the potential of these devices for applications in two-color high-temperature detectors and solar cells.

## 2. Materials and Methods

The overall design of the studied diodes and their fabrication procedure are very similar to those reported in our previous publication [17]. As before, the p-ZnTe/n-CdTe diodes containing PbTe quantum dots (QDs) were grown by molecular beam epitaxy (MBE) on a semi-insulating GaAs (100) substrate. The molecular fluxes of elemental Pb, Cd, Te, and Zn were supplied by standard effusion cells containing ultra-pure (99.99999%) elements. The oxide-protecting layer was removed from the GaAs substrate surface by a standard thermal annealing process. In the first stage of the MBE growth, a 2.8 µm-thick p-type ZnTe buffer layer was deposited. Nitrogen acceptor doping was achieved using a radio-frequency nitrogen plasma source. In the second stage, the depleted charge region of the diode was formed by the growth of an undoped PbTe/CdTe multilayer structure. This structure consisted of 20 repetitions of 13 nm-thick PbTe layers separated by 21 nm-thick CdTe barriers. To transform the thin PbTe layers into rows of quantum dots, the structure was annealed for 10 min at the growth temperature of 350 °C. The choice of 350 °C follows prior systematic studies [8,9,10,11] and our own preliminary tests in the 300–400 °C range. Lower annealing temperatures (≤320 °C) resulted in incomplete QD formation, while temperatures above 370 °C led to QD coarsening and reduced uniformity. In the final stage of the MBE growth, the structure was capped with a 0.38 µm-thick n-type CdTe layer. N-type conductivity was achieved by doping this layer with iodine, supplied from a solid ZnI_2_ source. The CdTe layers were deposited at a slight overpressure of Cd, with beam equivalent pressures (BEPs) of 1.1 × 10^−6^ mbar for Cd and 1.0 × 10^−6^ mbar for Te. On the other hand, the epitaxy of PbTe and ZnTe requires Te-rich conditions, so the BEP for Pb flux was set at 6.0 × 10^−7^ and for Zn at 5.0 × 10^−7^ mbar. The MBE process was monitored in situ by reflection high-energy electron diffraction (RHEED), which revealed sharp streaky patterns throughout growth, indicating high-quality structures. The carrier concentrations in the n-type and p-type layers were estimated to be in the range of 1–3 × 10^19^ cm^−3^. To enable electrical contact to the internal p-type ZnTe layer, a mesa structure was defined by etching with a methanol–bromine solution. Electrical contacts were subsequently formed on both sides of the diodes using indium soldering. The contact resistance, estimated from four-contact probe measurements, is in the order of 1 Ω for the p- and n-sides.

Cross-sectional imaging and electron beam-induced current (EBIC) measurements of the p-ZnTe/n-CdTe diodes containing PbTe quantum dots were performed using a ZEISS EVO HD15 scanning electron microscope (SEM) equipped with a Digital Image Scanning System 5 EBIC setup (point electronic GmbH, Halle, Germany). An accelerating voltage of 20 kV and a beam current of 500 pA were applied. Under these conditions, the beam spot size is 20–30 nm. EBIC signals were recorded over a temperature range of 60–290 K. SEM imaging was used to examine the overall diode structure and to determine the precise thickness of individual layers. For these measurements, the structures were cleaved along the growth direction ([100] axis), and scans were acquired in the (011) planes, which correspond to the preferred cleavage planes of zinc-blende II–VI crystals.

The measurements of photo-sensitivity in the infrared were performed using an infrared spectrometer comprising an M150 monochromator (Solar Laser System, Minsk, Beloruss), a Nernst lamp as the infrared light source, and a Thorlabs mechanical chopper operating at 730 Hz. The output signal was detected using a MFLI 500 kHz/5 MHz Lock-in Amplifier (Zurich Instruments, Zurich, Swiss).

## 3. Results and Discussion

**Figure 1:**
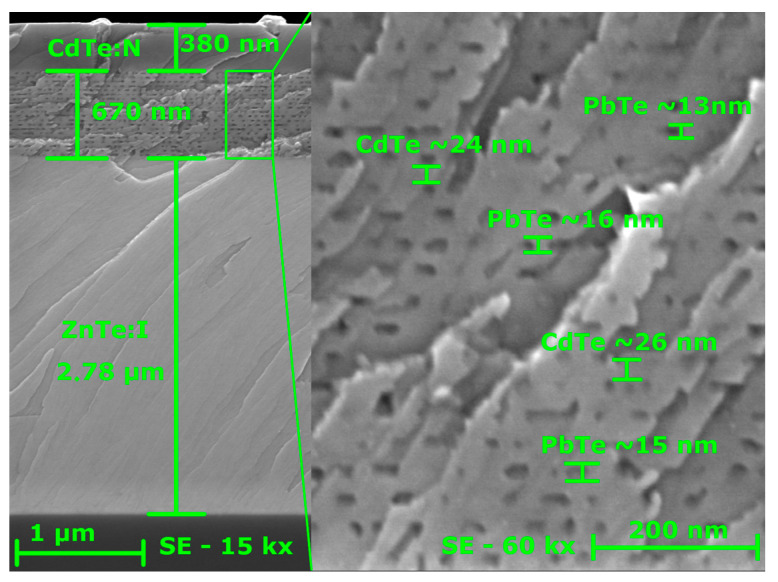
Cross-sectional SEM micrographs of a p-ZnTe/n-CdTe diode with integrated PbTe QDs. (**Left panel**): Full device stack, comprising a 2.8 µm p-type ZnTe epitaxial layer, a PbTe/CdTe multilayer region, and a 0.38 µm n-type CdTe layer. The dark contrast region at the bottom corresponds to the GaAs substrate. (**Right panel**): High-magnification SEM image of the PbTe/CdTe multilayer region, demonstrating that under the applied epitaxial growth conditions, the nominally two-dimensional PbTe layers undergo lateral self-organization into rows of PbTe quantum dots.

The left panel of Figure 1 depicts a cross-sectional SEM image of the diode, highlighting its layered structure. Horizontal stripes of varying contrast clearly delineate the GaAs substrate, the p-type ZnTe layer, the PbTe/CdTe multilayer region, and the n-type CdTe cap. The layer thicknesses determined from the SEM image—2.8, 0.68, and 0.38 µm, respectively—are in good agreement with the values expected from the MBE growth parameters. The right panel of Figure 1 shows the PbTe/CdTe multilayer region at higher magnification. Rows of PbTe QDs separated by CdTe barriers are distinctly visible. The average dimensions of the QDs are approximately 9–16 nm in height and 25–35 nm in length, with adjacent QDs rows spaced by about 21 nm. Because of the lattice-structure-type mismatch between PbTe (rock-salt structure) and CdTe and ZnTe (zinc blende structure), the materials are immiscible. As a result, there is no intermixing between the QD material and the surrounding matrix, and the QDs are formed from pure PbTe [8,9,10,11].

**Figure 2:**
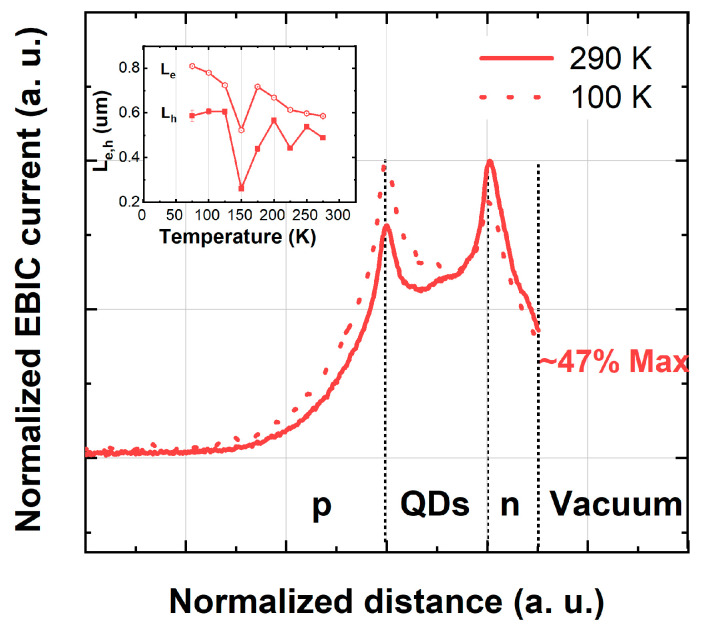
Cross-sectional electron beam-induced current (EBIC) scans measured at 100 and 290 K. Two distinct maxima in the EBIC profiles mark the interfaces between the p-ZnTe layer, the PbTe quantum-dot–containing region, and the n-CdTe layer. To facilitate comparison of temperature-dependent behavior, all EBIC curves are normalized to their respective maximum values—inset: temperature dependence of the minority-carrier diffusion length extracted from the EBIC line scans.

Figure 2 shows the electron beam-induced current (EBIC) scan across the structure. The EBIC signal measured at the p-n junction reaches its maximum at the point where the built-in electric field is strongest. The EBIC maximum, therefore, indicates the location of the p-n junction in the material. In the case of our diodes, two EBIC signal maxima are visible, as shown in Figure 2. The first maximum occurs at the boundary between the p-ZnTe and PbTe/CdTe layers. In contrast, the second maximum occurs at the boundary between the PbTe/CdTe and n-CdTe layers. The appearance of this double maximum indicates that the PbTe/CdTe layer, i.e., the layer containing PbTe quantum dots, is quasi-insulating and therefore forms two connected p-n junctions, one with the p-ZnTe layer and the other with the n-CdTe layer.

The minority carrier diffusion lengths (L_e_ and L_h_) for electrons and holes are shown in the inset of Figure 2, extracted from the EBIC scans according to the following relation: I_EBIC_ = I_0_ exp(−x/L_e,h_), where I_EBIC_ is the EBIC signal, I_0_ is a constant, x describes the position of the generating electron beam. The diffusion lengths obtained from the EBIC scans are relatively short (L_e_ = 0.7–0.8 μm, L_h_ = 0.5–0.6 μm) and exhibit only a weak temperature dependence. Shorter, temperature-independent diffusion lengths were also observed in our previous study [17]. To enhance the collection of photoexcited carriers at the metallic contacts—and thus improve the light sensitivity of the diodes—the n-type cap layer was made significantly thinner (0.38 µm) than in the previous study (0.92 µm). Due to the thinner cap, the electron beam–induced current at the metallic contact reaches 47% of its maximum value, compared to only a few percent for the thicker cap.

**Figure 3:**
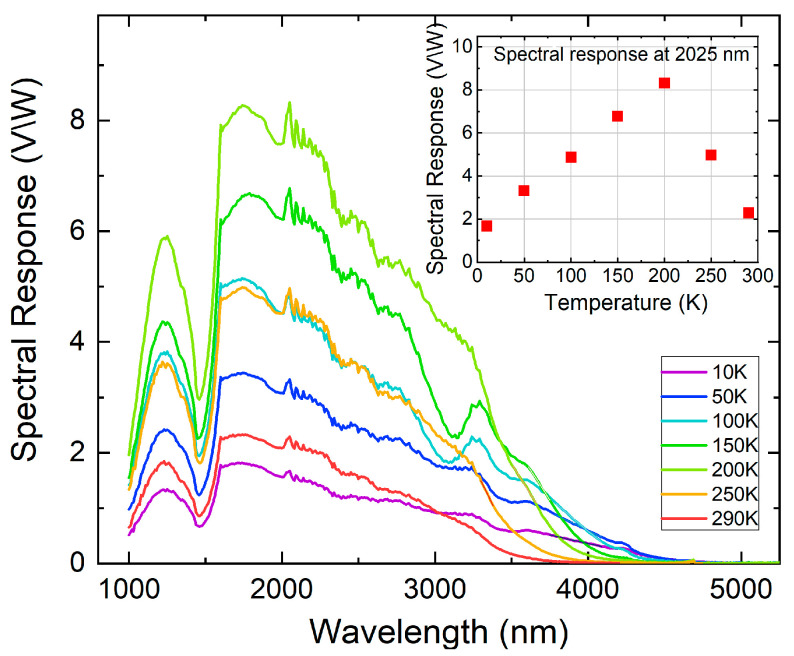
Infrared spectral response of a p-ZnTe/n-CdTe diode with integrated PbTe QDs at different temperatures in the range of 10–290 K. Inset: Temperature dependence of the maximal photo-response signal at 2.025 µm.

The typical photosensitivity spectra of the p-ZnTe/n-CdTe diode with integrated PbTe QDs, measured in the infrared (IR) spectral region at temperatures from 10 to 290 K, are shown in Figure 3. During optical measurements, illumination was applied through the n-type CdTe cap layer. The diodes exhibit a pronounced photo-response to infrared radiation, persisting up to room temperature. As shown in the inset to Figure 3, the photosensitivity increases significantly with decreasing temperature, reaching a maximum at T = 200 K. Further decreases in temperature result in a reduction in signal intensity. The maximum sensitivity, observed at 2.0 µm and 200 K, is 8 V/W.

Notably, the sensitivity of the diodes incorporating QDs increased by nearly an order of magnitude compared to closely related devices containing single- or multi-layer PbTe nano-inclusions, as reported in our previous work [17]. This enhancement is likely associated with two main factors. First, in QD-based diodes, a substantial fraction of the electrons traversing the p–n junction can bypass the PbTe nano-inclusions and transition from the p-type to the n-type region through the intrinsic CdTe layer. This transport pathway is unavailable in structures where PbTe nano-inclusions form single or multiple layers oriented perpendicular to the electron flow. In those configurations, electrons become trapped in the potential wells of the PbTe layers, and their subsequent transport requires tunneling across the PbTe–CdTe interfaces. Second, as mentioned above, the increased photosensitivity can be attributed to the thinner n-type cap layer. Because of this reduced thickness, and since the n-type cap is now twice as thin as the hole carrier diffusion length, a larger proportion of photoexcited holes can reach the diode’s surface contact (see Figure 2), increasing the device’s sensitivity.

Temperature affects not only the intensity of the photoresponse but also its spectral shape, especially in the long-wavelength region. At 290 K, the signal drops around 3.7 μm, while at 10 K, the infrared response extends up to about 4.5 μm. The cut-off energies increase linearly with temperature, showing that the mid-infrared sensitivity of the broadband p-ZnTe/n-CdTe diodes results from PbTe quantum dots. The observed blue shift in the measured cutoff energies relative to the PbTe energy gap is due to quantum confinement. Using the effective mass approximation for spherical PbTe quantum dots (m_e_ ≈ 0.043 m_0_; m_h_ ≈ 0.051 m_0_), we calculated the expected increase in the energy gap caused by confinement for quantum dots with dimensions corresponding to the measured average size. The calculated confinement shift of about 70 meV is consistent with the experimentally observed blue-shifted cutoff relative to the energy gap.

**Figure 4:**
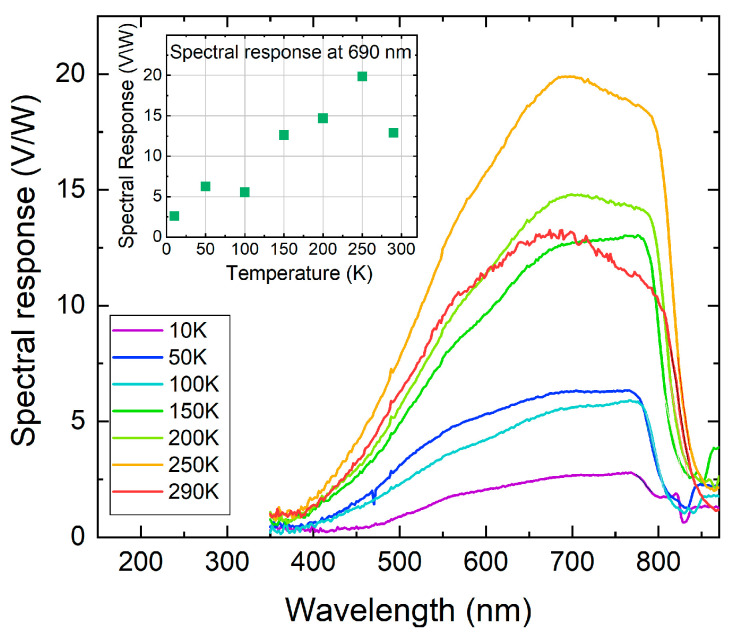
Spectral response of a p-ZnTe/n-CdTe diode with integrated PbTe QDs in the visible spectral range at different temperatures in the range of 10–290 K. Inset: Temperature dependence of the maximal photo-response signal at 0.69 µm.

The diodes also show sensitivity to radiation in the visible spectrum, as illustrated in Figure 4. For optical characterization in the visible spectral range, a USHIO 12 V 100 W Xenon lamp coupled with a monochromator was used. ZnTe and CdTe absorption dominate the visible-range response, while PbTe QDs contribute marginally due to their mid-infrared bandgap. In the visible range, the photoresponsivity signal is stronger than in IR, reaching 20 V/W at a wavelength of around 0.7 µm and a slightly higher temperature of 250 K. The spectra shown in Figure 4 are almost identical to the quantum efficiency spectra of the p-ZnTe/n-CdTe solar cell without PbTe quantum dots presented in ref. [18] (Figure 1), A comparison of these results clearly shows that the diode without PbTe QDs exhibits only the intrinsic ZnTe/CdTe response. In contrast, the diode with integrated PbTe QDs exhibits an additional narrow-band IR response, confirming the functional role of quantum dots.

Considering the lock-in amplifier load resistance of 1 MΩ, the zero-bias resistance of the diode of approximately 10 kΩ, and the diode active area of 1.16 mm^2^, the estimated detectivities of the device are relatively low both in the infrared and visible spectral ranges, amounting to 7 × 10^7^ cm·Hz^1/2^/W and to 2 × 10^8^ cm·Hz^1/2^/W, respectively. Although the detectivity values are lower than those of optimized infrared and visible light detectors, the p-ZnTe/n-CdTe diodes with integrated PbTe QDs exhibit a broad spectral response—from the visible to the mid-infrared, and they can operate up to room temperature. These characteristics make the devices a promising candidate for broadband sensing and multispectral photodetection applications.

However, many other key optimization factors that can improve the performance of p-ZnTe/n-CdTe detectors with embedded PbTe QDs must be considered and experimentally optimized. These factors include: QD density to balance absorption and carrier transport; annealing time to reduce interfacial traps; acceptor and donor dopant concentrations to adjust the depletion region; and interfacial passivation to inhibit recombination, etc. The long-term stability of these detectors also needs to be investigated.

## 4. Summary

Incorporating narrow-band PbTe quantum dots into the depletion region of p-ZnTe/n-CdTe diodes enables infrared sensitivity up to room temperature without compromising device performance. Relatively short minority-carrier diffusion lengths measured by EBIC suggest that responsivity could be improved by reducing the distance between the depletion layer and the contacts. These findings highlight the potential of such devices for high-temperature infrared detection and dual-color solar cell applications.

## Data Availability

The original contributions presented in this study are included in the article. Further inquiries can be directed to the corresponding author.
